# Supplementary material for: Short-term impact of preschool sound exposure on outer hair cell function in young children: An analysis using pressurised distortion product otoacoustic emissions
Source: PLoS One. 2025 Nov 21;20(11):e0332863. doi: 10.1371/journal.pone.0332863 (PMC12637916; doi:10.1371/journal.pone.0332863)
Supplement: S1 File — (PDF) [file pone.0332863.s001.pdf]

S1 Supporting Tables

Data Inclusion Criteria

Table A. Stepwise data inclusion criteria for pDPOAE Measurements

	Frequency	Total Measurements	Pass Tympanometry	SNR ≥ 6 dB	Amplitude ≥ -10 dB	Reliability ≥ 80%
RIGHT EAR	3 kHz	238	160	159	159	159
	4 kHz	238	160	158	158	157
	5 kHz	238	160	159	158	146
	6 kHz	238	160	160	158	137
	7 kHz	239	160	158	157	146
	8 kHz	238	160	160	150	148
LEFT EAR	3 kHz	235	147	145	145	144
	4 kHz	235	147	145	145	145
	5 kHz	235	147	145	145	132
	6 kHz	235	147	145	145	125
	7 kHz	234	147	145	144	124
	8 kHz	230	142	132	109	102

Note. This table presents the stepwise data inclusion criteria for pDPOAE measurements in both ears across tested frequencies (3–8 kHz). The total number of measurements at each frequency is listed, along with the number of measurements retained after applying successive quality control criteria: passing tympanometry, signal-to-noise ratio (SNR) ≥ 6 dB, amplitude ≥ -10 dB, and reliability ≥ 80%. The final column represents the number of measurements meeting all criteria.

Table B. Number of measured and retained pDPOAE responses by timepoint and weekday.

Weekday	Timepoint	Measured pDPOAE *n*	Post inclusion pDPOAE *n*	% Retained
Beginning of week	Morning	810	457	56.4
	Afternoon	778	446	57.3
End of week	Morning	797	375	47.1
	Afternoon	767	350	45.6

Note. This table shows the number of measured pDPOAE responses before and after applying predefined quality inclusion criteria (tympanometry within normal limits, SNR ≥ 6 dB, amplitude ≥ -10 dB SPL, and reliability ≥ 80%) across the four measurement sessions. The percentage retained reflects the proportion of valid measurements relative to the total number collected at each timepoint.

Table C. Number of measured and retained pDPOAE responses by timepoint and weekday.

Ear	Category	Percent	n
Right	≥ to -50 daPa	39.7	628
	-49 to -1 daPa	46.8	740
	0 to +50 daPa	13.5	213
Left	≥ to -50 daPa	40.6	609
	-49 to -1 daPa	46.7	700
	0 to +50 daPa	12.7	191

Note. This table presents the distribution of tympanometric peak pressures for all retained pDPOAE measurements, grouped by ear and pressure range. Values reflect the number and percentage of measurements falling within each pressure category: ≥ –50 daPa, –49 to –1 daPa, and 0 to +50 daPa. Nearly half of all retained responses fell within the –49 to –1 daPa range for both ears.

RESULTS

Table D Descriptive statistics for DPOAE amplitudes (dB SPL) across frequencies (3–8 kHz) for the left and right ears, including mean, minimum (Min), maximum (Max), and standard deviation (SD).

	Frequency	Mean (dB SPL)	Min (dB SPL)	Max (dB SPL)	SD (dB SPL)
RIGHT	3 kHz	7.6	-9.3	17.6	5.9
	4 kHz	9.1	-4.2	19.9	4.8
	5 kHz	9.5	-8.6	18	4.2
	6 kHz	9.9	-5	18.9	4.9
	7 kHz	8.2	-7.8	17.8	5.1
	8 kHz	8.7	-6.9	18.2	4.7
LEFT	3 kHz	6.6	-9.2	19.3	5.8
	4 kHz	8.6	-8.3	18.2	5.1
	5 kHz	9	-7.5	18.7	4.6
	6 kHz	9.4	-7.1	19.1	4.8
	7 kHz	5.6	-9.5	19.8	6.1
	8 kHz	6.5	-8.4	18.5	5.2

Note. Descriptive statistics for distortion product otoacoustic emission (DPOAE) amplitudes in decibels sound pressure level (dB SPL) across frequencies (3–8 kHz) for both ears. The table presents the mean, minimum, maximum, and standard deviation (SD). Negative values indicate noise floor levels or reduced DPOAE signal detection.

Table E. Linear mixed-effects models assessing the effects of L_{AeqTi}, day of the week, and sex on DPOAE amplitudes (3–8 kHz).

Ear	DPOAE Amplitude	Model	Fixed Effect	*β*	CI 95% Low	CI 95% High	SE	*p*	ICC	AIC	BIC
RIGHT EAR	3 kHz	Base	Intercept	7.573	4.32	6.84	0.85	0.000	0.87	416.02	422.76
		Model 1	L_{AeqTi}	0.125	-0.10	0.35	0.11	0.244	0.84	419.18	430.42
			Day of week	-0.696	-1.95	0.54	0.63	0.280	0.84	419.18	430.42
		Model 2	L_{AeqTi}	0.116	-0.10	0.34	0.11	0.282	0.85	416.95	430.44
			Day of week	-0.708	-1.96	0.52	0.63	0.270	0.85	416.95	430.44
			Sex	-1.956	-5.25	1.31	1.68	0.251	0.85	416.95	430.44
	4 kHz	Base	Intercept	9.635	2.20	4.69	0.64	0.000	0.57	413.87	420.66
		Model 1	L_{AeqTi}	0.033	-0.18	0.24	0.11	0.754	0.55	418.87	430.18
			Day of week	-0.323	-1.94	1.31	0.82	0.698	0.55	418.87	430.18
		Model 2	L_{AeqTi}	0.046	-0.17	0.26	0.11	0.666	0.54	417.50	431.08
			Day of week	-0.312	-1.93	1.33	0.83	0.709	0.54	417.50	431.08
			Sex	1.307	-1.26	3.84	1.31	0.325	0.54	417.50	431.08
	5 kHz	Base	Intercept	9.783	1.62	4.58	0.65	0.000	0.52	377.74	384.27
		Model 1	L_{AeqTi}	-0.102	-0.31	0.10	0.11	0.335	0.53	381.55	392.42
			Day of week	0.513	-1.16	2.21	0.85	0.552	0.53	381.55	392.42
		Model 2	L_{AeqTi}	-0.082	-0.28	0.12	0.10	0.434	0.50	378.36	391.40
			Day of week	0.610	-1.06	2.34	0.86	0.483	0.50	378.36	391.40
			Sex	2.263	-0.36	4.86	1.35	0.101	0.50	378.36	391.40
	6 kHz	Base	Intercept	10.523	1.96	4.29	0.58	0.000	0.66	334.23	340.56
		Model 1	L_{AeqTi}	0.010	-0.19	0.20	0.09	0.914	0.64	339.98	350.53
			Day of week	0.200	-1.20	1.64	0.71	0.782	0.64	339.98	350.53
		Model 2	L_{AeqTi}	0.016	-0.18	0.21	0.09	0.861	0.63	339.10	351.77
			Day of week	0.188	-1.22	1.63	0.72	0.796	0.63	339.10	351.77
			Sex	0.983	-1.39	3.32	1.21	0.423	0.63	339.10	351.77

	7 kHz	Base	Intercept	8.157	3.35	5.90	0.76	0.000	0.79	379.86	386.39
		Model 1	L_{AeqTi}	0.139	-0.08	0.35	0.11	0.200	0.75	383.33	394.20
			Day of week	0.585	-0.83	2.05	0.72	0.427	0.75	383.33	394.20
		Model 2	L_{AeqTi}	0.134	-0.08	0.35	0.11	0.220	0.76	382.52	395.57
			Day of week	0.578	-0.83	2.05	0.72	0.432	0.76	382.52	395.57
			Sex	-0.488	-3.51	2.53	1.55	0.755	0.76	382.52	395.57
	8 kHz	Base	Intercept	3.034	4.65	7.56	0.97	0.003	0.85	407.39	413.96
		Model 1	L_{AeqTi}	0.229	-0.02	0.48	0.13	0.082	0.84	408.45	419.40
			Day of week	0.903	-0.53	2.35	0.73	0.229	0.84	408.45	419.40
		Model 2	L_{AeqTi}	0.222	-0.03	0.48	0.13	0.094	0.84	407.01	420.15
			Day of week	0.896	-0.54	2.35	0.73	0.233	0.84	407.01	420.15
			Sex	-1.046	-4.80	2.72	1.93	0.592	0.84	407.01	420.15
**LEFT EAR**	3 kHz	Base	Intercept	7.379	3.94	6.71	0.87	0.000	0.81	381.20	387.63
		Model 1	L_{AeqTi}	0.118	-0.13	0.36	0.13	0.355	0.80	383.95	394.66
			Day of week	-0.792	-2.33	0.71	0.77	0.314	0.80	383.95	394.66
		Model 2	L_{AeqTi}	0.120	-0.12	0.37	0.13	0.344	0.80	382.16	395.02
			Day of week	-0.802	-2.34	0.70	0.77	0.308	0.80	382.16	395.02
			Sex	-1.588	-5.05	1.87	1.78	0.377	0.80	382.16	395.02
	4 kHz	Base	Intercept	8.836	2.01	4.44	0.63	0.000	0.57	353.40	359.78
		Model 1	L_{AeqTi}	0.075	-0.14	0.29	0.11	0.499	0.55	358.08	368.72
			Day of week	-0.039	-1.68	1.61	0.84	0.963	0.55	358.08	368.72
		Model 2	L_{AeqTi}	0.070	-0.13	0.27	0.11	0.512	0.51	352.94	365.70
			Day of week	-0.001	-1.62	1.63	0.83	0.999	0.51	352.94	365.70
			Sex	2.782	0.40	5.17	1.23	**0.030**[*]	0.51	352.94	365.70
	5 kHz	Base	Intercept	8.643	0.00	6.18	0.90	0.000	0.49	351.77	357.79
		Model 1	L_{AeqTi}	-0.022	-0.33	0.29	0.16	0.889	0.47	353.51	363.54
			Day of week	1.694	-0.89	4.41	1.33	0.215	0.47	353.51	363.54

	Model 2	L_{AeqTi}	-0.035	-0.34	0.27	0.16	0.824	0.46	350.10	362.15
		Day of week	1.762	-0.79	4.50	1.32	0.196	0.46	350.10	362.15
		Sex	2.838	-0.72	6.41	1.85	0.135	0.46	350.10	362.15
6 kHz	Base	Intercept	8.886	3.15	6.74	0.97	0.000	0.60	368.98	375.11
	Model 1	L_{AeqTi}	0.079	-0.23	0.39	0.16	0.624	0.60	368.66	378.88
		Day of week	2.405	0.04	4.86	1.22	0.058	0.60	368.66	378.88
	Model 2	L_{AeqTi}	0.077	-0.23	0.39	0.16	0.634	0.61	366.78	379.04
		Day of week	2.387	0.05	4.83	1.21	0.059	0.61	366.78	379.04
		Sex	1.658	-2.11	5.44	1.96	0.402	0.62	365.79	378.04
7 kHz	Base	Intercept	5.807	4.84	8.36	1.10	0.000	0.85	348.83	354.80
	Model 1	L_{AeqTi}	0.099	-0.25	0.45	0.17	0.572	0.85	351.32	361.26
		Day of week	1.031	-0.86	3.04	0.97	0.306	0.85	351.32	361.26
	Model 2	L_{AeqTi}	0.106	-0.24	0.45	0.18	0.550	0.86	349.76	361.69
		Day of week	1.017	-0.86	3.03	0.97	0.312	0.86	349.76	361.69
		Sex	0.642	-3.77	5.12	2.29	0.781	0.86	349.76	361.69
8 kHz	Base	Intercept	3.426	4.17	7.97	1.17	0.006	0.79	276.22	281.50
	Model 1	L_{AeqTi}	0.122	-0.24	0.48	0.18	0.514	0.78	278.71	287.52
		Day of week	0.932	-1.43	3.36	1.21	0.452	0.78	278.71	287.52
	Model 2	L_{AeqTi}	0.115	-0.24	0.47	0.19	0.543	0.78	276.92	287.48
		Day of week	0.942	-1.43	3.40	1.22	0.451	0.78	276.92	287.48
		Sex	-1.077	-5.71	3.66	2.43	0.660	0.78	276.92	287.48

Note. This table presents results from linear mixed-effects models assessing the effects of L_{AeqTi}, day of the week, and sex on afternoon DPOAE amplitudes (3–8 kHz). β represents the estimated effect size, with 95% confidence intervals (CI), standard errors (SE), and intraclass correlation coefficients (ICC). Model fit is evaluated using the Akaike Information Criterion (AIC) and Bayesian Information Criterion (BIC). P-values= **>0.05***

Table F. Linear mixed-effects models assessing the effects of $L_{AFmax,95}$, day of the week, and Sex on DPOAE amplitudes (3–8 kHz).

Ear	DPOAE Amplitude	Model	Fixed Effect	*β*	CI 95% Low	CI 95% High	SE	*p*	ICC	AIC	BIC
RIGHT EAR	3 kHz	Base	Intercept	7.573	4.32	6.84	0.85	0.000	0.87	416.02	422.76
		Model 1	$L_{AFmax,95}$	0.234	-0.02	0.49	0.13	0.071	0.85	416.80	428.04
			Day of week	-0.782	-2.02	0.41	0.61	0.214	0.85	416.80	428.04
		Model 2	$L_{AFmax,95}$	0.220	-0.03	0.48	0.13	0.092	0.85	414.79	428.28
			Day of week	-0.786	-2.02	0.40	0.61	0.210	0.85	414.79	428.28
			Sex	-1.790	-5.05	1.45	1.67	0.290	0.85	414.79	428.28
	4 kHz	Base	Intercept	9.635	2.20	4.69	0.64	0.000	0.57	413.87	420.66
		Model 1	$L_{AFmax,95}$	0.108	-0.13	0.34	0.12	0.372	0.56	417.87	429.19
			Day of week	-0.363	-1.96	1.24	0.81	0.658	0.56	417.87	429.19
		Model 2	$L_{AFmax,95}$	0.126	-0.11	0.36	0.12	0.304	0.55	416.32	429.89
			Day of week	-0.360	-1.96	1.25	0.81	0.661	0.55	416.32	429.89
			Sex	1.426	-1.14	3.97	1.32	0.286	0.55	416.32	429.89
	5 kHz	Base	Intercept	9.783	1.62	4.58	0.65	0.000	0.52	377.74	384.27
		Model 1	$L_{AFmax,95}$	-0.033	-0.27	0.21	0.12	0.786	0.51	382.12	392.99
			Day of week	0.553	-1.15	2.28	0.87	0.529	0.51	382.12	392.99
		Model 2	$L_{AFmax,95}$	-0.009	-0.24	0.23	0.12	0.942	0.49	378.68	391.72
			Day of week	0.642	-1.05	2.39	0.87	0.465	0.49	378.68	391.72
			Sex	2.363	-0.25	4.97	1.35	0.089	0.49	378.68	391.72
	6 kHz	Base	Intercept	10.523	1.96	4.29	0.58	0.000	0.66	334.23	340.56
		Model 1	$L_{AFmax,95}$	0.157	-0.05	0.37	0.11	0.142	0.67	337.53	348.08
			Day of week	0.218	-1.13	1.58	0.68	0.752	0.67	337.53	348.08
		Model 2	$L_{AFmax,95}$	0.167	-0.04	0.38	0.11	0.122	0.67	336.42	349.08
			Day of week	0.206	-1.14	1.56	0.68	0.765	0.67	336.42	349.08
			Sex	1.142	-1.20	3.48	1.21	0.352	0.67	336.42	349.08
	7 kHz	Base	Intercept	8.157	3.35	5.90	0.76	0.000	0.79	379.86	386.39
		Model 1	$L_{AFmax,95}$	0.153	-0.10	0.40	0.13	0.241	0.77	383.15	394.02
			Day of week	0.468	-0.92	1.90	0.71	0.516	0.77	383.15	394.02
		Model 2	$L_{AFmax,95}$	0.148	-0.10	0.40	0.13	0.261	0.77	382.32	395.37

			Day of week	0.465	-0.92	1.90	0.71	0.519	0.77	382.32	395.37
			Sex	-0.483	-3.54	2.57	1.57	0.761	0.77	382.32	395.37
	8 kHz	Base	Intercept	3.034	4.65	7.56	0.97	0.003	0.85	407.39	413.96
		Model 1	$L_{AFmax,95}$	0.199	-0.10	0.50	0.15	0.194	0.83	409.53	420.48
			Day of week	0.748	-0.70	2.21	0.74	0.320	0.83	409.53	420.48
		Model 2	$L_{AFmax,95}$	0.189	-0.11	0.49	0.15	0.222	0.84	408.06	421.19
			Day of week	0.745	-0.71	2.21	0.74	0.323	0.84	408.06	421.19
			Sex	-1.077	-4.87	2.72	1.95	0.584	0.84	408.06	421.19
LEFT EAR	3 kHz	Base	Intercept	7.379	3.94	6.71	0.87	0.000	0.81	381.20	387.63
		Model 1	$L_{AFmax,95}$	0.009	-0.29	0.31	0.15	0.955	0.81	384.44	395.16
			Day of week	-0.901	-2.43	0.58	0.76	0.248	0.81	384.44	395.16
		Model 2	$L_{AFmax,95}$	0.006	-0.29	0.31	0.15	0.970	0.81	382.70	395.55
			Day of week	-0.912	-2.44	0.56	0.76	0.241	0.81	382.70	395.55
			Sex	-1.542	-5.05	1.95	1.80	0.398	0.81	382.70	395.55
	4 kHz	Base	Intercept	8.836	2.01	4.44	0.63	0.000	0.57	353.40	359.78
		Model 1	$L_{AFmax,95}$	-0.037	-0.29	0.21	0.12	0.768	0.57	358.22	368.86
			Day of week	-0.117	-1.74	1.50	0.82	0.887	0.57	358.22	368.86
		Model 2	$L_{AFmax,95}$	-0.027	-0.27	0.21	0.12	0.820	0.53	353.09	365.86
			Day of week	-0.073	-1.67	1.54	0.82	0.929	0.53	353.09	365.86
			Sex	2.807	0.39	5.22	1.25	**0.030***	0.53	353.09	365.86
	5 kHz	Base	Intercept	8.643	0.00	6.18	0.90	0.000	0.49	351.77	357.79
		Model 1	$L_{AFmax,95}$	-0.186	-0.53	0.15	0.17	0.286	0.49	352.20	362.24
			Day of week	1.577	-0.95	4.24	1.29	0.236	0.49	352.20	362.24
		Model 2	$L_{AFmax,95}$	-0.193	-0.52	0.13	0.17	0.262	0.48	348.71	360.75
			Day of week	1.650	-0.84	4.33	1.29	0.213	0.48	348.71	360.75
			Sex	2.887	-0.65	6.44	1.84	0.127	0.48	348.71	360.75
	6 kHz	Base	Intercept	8.886	3.15	6.74	0.97	0.000	0.60	368.98	375.11
		Model 1	$L_{AFmax,95}$	-0.180	-0.54	0.18	0.18	0.329	0.62	367.68	377.90
			Day of week	2.257	-0.05	4.66	1.19	0.068	0.62	367.68	377.90
		Model 2									

		$L_{AFmax,95}$	-0.180	-0.54	0.17	0.18	0.330	0.62	365.79	378.04
		Day of week	2.243	-0.04	4.64	1.19	0.069	0.62	365.79	378.04
		Sex	1.658	-2.11	5.44	1.96	0.402	0.62	365.79	378.04
7 kHz	Base	Intercept	5.807	4.84	8.36	1.10	0.000	0.85	348.83	354.80
	Model 1	L $L_{AFmax,95}$	-0.113	-0.51	0.28	0.20	0.574	0.85	351.04	360.99
		Day of week	1.000	-0.91	3.03	0.98	0.323	0.85	351.04	360.99
	Model 2	$L_{AFmax,95}$	-0.114	-0.50	0.28	0.20	0.573	0.85	349.52	361.45
		Day of week	0.987	-0.91	3.02	0.98	0.329	0.85	349.52	361.45
		Sex	0.533	-3.87	4.96	2.28	0.816	0.85	349.52	361.45
8 kHz	Base	Intercept	3.426	4.17	7.97	1.17	0.006	0.79	276.22	281.50
	Model 1	$L_{AFmax,95}$	0.036	-0.43	0.48	0.22	0.870	0.78	278.77	287.58
		Day of week	0.842	-1.55	3.39	1.23	0.503	0.78	278.77	287.58
	Model 2	$L_{AFmax,95}$	0.031	-0.43	0.48	0.22	0.892	0.78	276.93	287.49
		Day of week	0.854	-1.53	3.45	1.24	0.501	0.78	276.93	287.49
		Sex	-1.187	-5.83	3.53	2.43	0.629	0.78	276.93	287.49

Note. This table presents results from linear mixed-effects models assessing the effects of $L_{AFmax,95,}$day of the week, and sex on afternoon DPOAE amplitudes (3–8 kHz). β represents the estimated effect size, with 95% confidence intervals (CI), standard errors (SE), and intraclass correlation coefficients (ICC). Model fit is evaluated using the Akaike Information Criterion (AIC) and Bayesian Information Criterion (BIC). P-values= >0.05*

Effects Time-of-Day and End-of-Week Variability in DPOAE Amplitudes

Table G. Linear mixed-effects models assessing the effects of Time: Morning vs. Afternoon and Day; Beginning of week vs. End of week of the week, and sex on DPOAE amplitudes

Ear	DPOAE Amplitude	Model	Fixed Effect	β	CI 95% Low	CI 95% High	SE	p	ICC	AIC	BIC
RIGHT EAR	3 kHz	Base	Intercept	8.015	6.55	9.48	0.74	0.000	0.75	908.01	920.29
		Model 1	Time	-0.358	-1.28	0.56	0.46	0.441	0.75	908.01	920.29
		Model 2	Time	-0.359	-1.28	0.56	0.47	0.442	0.75	909.54	924.89
			Day	-0.013	-1.01	0.99	0.51	0.980	0.75	909.54	924.89
		Model 3	Time	-0.112	-1.35	1.12	0.62	0.858	0.75	909.48	927.89
			Day	0.270	-1.10	1.64	0.69	0.697	0.75	909.48	927.89
			Time x Day	-0.564	-2.42	1.29	0.94	0.548	0.75	909.48	927.89
		Model 4	Time	0.320	-1.53	2.17	0.93	0.733	0.74	904.50	935.19
			Day	0.088	-2.08	2.26	1.10	0.936	0.74	904.50	935.19
			Sex	-1.855	-5.02	1.31	1.59	0.247	0.74	904.50	935.19
			Time x Day	-0.104	-3.07	2.86	1.50	0.945	0.74	904.50	935.19
	4 kHz	Base	Intercept	8.880	7.61	10.15	0.64	0.000	0.65	885.25	897.47
		Model 1	Time	0.564	-0.40	1.52	0.48	0.247	0.65	885.25	897.47
		Model 2	Time	0.572	-0.39	1.54	0.49	0.243	0.65	886.48	901.76
			Day	0.262	-0.76	1.29	0.52	0.613	0.65	886.48	901.76
		Model 3	Time	1.327	0.03	2.62	0.65	**0.045***	0.65	883.75	902.09
			Day	1.119	-0.30	2.53	0.71	0.120	0.65	883.75	902.09
			Time x Day	-1.661	-3.57	0.25	0.96	0.087	0.65	883.75	902.09
		Model 4	Time	1.706	-0.23	3.64	0.98	0.083	0.64	879.53	910.09
			Day	1.315	-0.90	3.53	1.12	0.242	0.64	879.53	910.09
			Sex	2.228	-0.61	5.07	1.43	0.123	0.64	879.53	910.09
			Time x Day	-1.336	-4.39	1.72	1.54	0.388	0.64	879.53	910.09
	5 kHz	Base	Intercept	9.350	8.09	10.61	0.63	0.000	0.68	805.16	817.09
		Model 1	Time	-0.076	-1.00	0.85	0.47	0.871	0.68	805.16	817.09
		Model 2	Time	-0.055	-0.98	0.87	0.47	0.907	0.67	805.69	820.61
			Day	0.505	-0.47	1.48	0.49	0.305	0.67	805.69	820.61
		Model 3									

		Time	0.120	-1.15	1.38	0.64	0.851	0.67	805.83	823.73
		Day	0.704	-0.67	2.08	0.69	0.311	0.67	805.83	823.73
		Time x Day	-0.378	-2.24	1.49	0.94	0.689	0.67	805.83	823.73
	Model 4	Time	-0.027	-2.04	1.99	1.01	0.978	0.66	793.88	823.72
		Day	2.390	0.20	4.58	1.11	**0.033***	0.66	793.88	823.72
		Sex	2.915	0.17	5.66	1.38	**0.037***	0.66	793.88	823.72
		Time x Day	-0.129	-3.15	2.89	1.52	0.933	0.66	793.88	823.72
6 kHz	Base	Intercept	9.365	8.20	10.53	0.59	0.000	0.58	760.24	771.92
	Model 1	Time	0.991	-0.05	2.03	0.52	0.061	0.58	760.24	771.92
	Model 2	Time	0.993	-0.05	2.04	0.52	0.061	0.58	761.55	776.15
		Day	0.137	-0.95	1.22	0.55	0.804	0.58	761.55	776.15
	Model 3	Time	1.380	0.02	2.74	0.68	**0.047***	0.57	760.83	778.35
		Day	0.607	-0.91	2.13	0.76	0.430	0.57	760.83	778.35
		Time x Day	-0.930	-3.02	1.16	1.05	0.380	0.57	760.83	778.35
	Model 4	Time	1.316	-0.72	3.35	1.02	0.202	0.57	756.31	785.51
		Day	1.251	-1.09	3.59	1.18	0.292	0.57	756.31	785.51
		Sex	1.309	-1.34	3.96	1.34	0.330	0.57	756.31	785.51
		Time x Day	-0.139	-3.58	3.30	1.73	0.936	0.57	756.31	785.51
7 kHz	Base	Intercept	7.395	5.95	8.84	0.72	0.000	0.72	840.20	852.13
	Model 1	Time	0.494	-0.52	1.51	0.51	0.334	0.72	840.20	852.13
	Model 2	Time	0.500	-0.51	1.51	0.51	0.329	0.71	839.83	854.75
		Day	0.724	-0.37	1.82	0.55	0.191	0.71	839.83	854.75
	Model 3	Time	0.778	-0.57	2.12	0.68	0.254	0.71	839.55	857.46
		Day	1.054	-0.46	2.56	0.76	0.169	0.71	839.55	857.46
		Time x Day	-0.641	-2.68	1.40	1.03	0.534	0.71	839.55	857.46
	Model 4	Time	1.173	-0.89	3.23	1.04	0.261	0.71	833.53	863.36
		Day	1.807	-0.72	4.34	1.28	0.160	0.71	833.53	863.36
		Sex	1.451	-1.70	4.60	1.58	0.362	0.71	833.53	863.36
		Time x Day	0.232	-3.14	3.60	1.70	0.892	0.71	833.53	863.36
8 kHz	Base	Intercept	2.473	0.72	4.22	0.87	0.006	0.85	846.75	858.74
	Model 1	Time	0.258	-0.61	1.13	0.44	0.556	0.85	846.75	858.74
	Model 2	Time	0.267	-0.59	1.13	0.43	0.538	0.85	844.99	859.98
		Day	0.869	-0.06	1.80	0.47	0.066	0.85	844.99	859.98

		Model 3	Time	0.433	-0.75	1.61	0.59	0.468	0.85	845.28	863.26
			Day	1.056	-0.25	2.36	0.66	0.111	0.85	845.28	863.26
			Time x Day	-0.353	-2.08	1.37	0.87	0.685	0.85	845.28	863.26
		Model 4	Time	0.510	-1.22	2.24	0.87	0.560	0.85	842.81	872.78
			Day	0.929	-1.11	2.97	1.03	0.369	0.85	842.81	872.78
			Sex	0.135	-3.59	3.86	1.87	0.943	0.85	842.81	872.78
			Time x Day	0.407	-2.34	3.16	1.38	0.769	0.85	842.81	872.78
LEFT EAR	3 kHz	Base	Intercept	6.770	5.23	8.32	0.77	0.000	0.78	826.80	838.68
		Model 1	Time	1.152	0.18	2.12	0.49	**0.021***	0.78	826.80	838.68
		Model 2	Time	1.133	0.16	2.11	0.49	**0.024***	0.78	827.64	842.48
			Day	-0.416	-1.45	0.62	0.52	0.425	0.78	827.64	842.48
		Model 3	Time	1.271	-0.03	2.58	0.66	0.056	0.78	827.75	845.57
			Day	-0.265	-1.67	1.14	0.71	0.709	0.78	827.75	845.57
			Time x Day	-0.310	-2.25	1.62	0.97	0.751	0.78	827.75	845.57
		Model 4	Time	0.985	-1.05	3.02	1.02	0.339	0.77	820.23	849.92
			Day	0.729	-1.56	3.02	1.15	0.529	0.77	820.23	849.92
			Sex	-2.381	-5.71	0.95	1.68	0.159	0.77	820.23	849.92
			Time x Day	-0.381	-3.46	2.70	1.55	0.807	0.77	820.23	849.92
	4 kHz	Base	Intercept	8.580	7.33	9.83	0.63	0.000	0.70	787.81	799.66
		Model 1	Time	0.499	-0.43	1.43	0.47	0.288	0.70	787.81	799.66
		Model 2	Time	0.496	-0.44	1.43	0.47	0.294	0.70	789.36	804.18
			Day	-0.086	-1.06	0.89	0.49	0.861	0.70	789.36	804.18
		Model 3	Time	0.295	-0.96	1.55	0.63	0.641	0.70	789.45	807.22
			Day	-0.306	-1.64	1.03	0.67	0.650	0.70	789.45	807.22
			Time x Day	0.448	-1.40	2.29	0.93	0.631	0.70	789.45	807.22
		Model 4	Time	-1.566	-3.45	0.32	0.95	0.102	0.71	781.39	811.02
			Day	-0.804	-2.93	1.32	1.07	0.455	0.71	781.39	811.02
			Sex	-1.290	-4.09	1.51	1.41	0.363	0.71	781.39	811.02
			Time x Day	1.567	-1.30	4.43	1.44	0.280	0.71	781.39	811.02
	5 kHz	Base	Intercept	9.089	7.61	10.57	0.74	0.000	0.73	757.69	769.22
		Model 1	Time	-0.204	-1.26	0.85	0.53	0.702	0.73	757.69	769.22
		Model 2	Time	-0.191	-1.25	0.87	0.53	0.722	0.73	758.00	772.42

		Day	0.564	-0.56	1.69	0.57	0.321	0.73	758.00	772.42
	Model 3	Time	-0.602	-2.03	0.83	0.72	0.405	0.72	757.32	774.61
		Day	0.139	-1.36	1.64	0.75	0.854	0.72	757.32	774.61
		Time x Day	0.907	-1.20	3.02	1.06	0.395	0.72	757.32	774.61
	Model 4	Time	-1.177	-3.47	1.12	1.16	0.311	0.72	754.55	783.38
		Day	-0.144	-2.63	2.34	1.25	0.909	0.72	754.55	783.38
		Sex	0.088	-3.26	3.43	1.68	0.958	0.72	754.55	783.38
		Time x Day	1.546	-1.93	5.02	1.74	0.378	0.72	754.55	783.38
6 kHz	Base	Intercept	8.628	6.81	10.44	0.91	0.000	0.79	748.95	760.26
	Model 1	Time	-0.073	-1.25	1.11	0.59	0.903	0.79	748.95	760.26
	Model 2	Time	-0.051	-1.22	1.12	0.59	0.931	0.80	747.16	761.30
		Day	1.044	-0.16	2.25	0.60	0.088	0.80	747.16	761.30
	Model 3	Time	-0.146	-1.75	1.46	0.81	0.857	0.79	746.99	763.96
		Day	0.940	-0.75	2.63	0.85	0.272	0.79	746.99	763.96
		Time x Day	0.206	-2.12	2.53	1.17	0.860	0.79	746.99	763.96
	Model 4	Time	-1.641	-4.12	0.84	1.24	0.191	0.80	740.71	768.99
		Day	-0.684	-3.32	1.95	1.32	0.606	0.80	740.71	768.99
		Sex	-1.371	-5.38	2.64	2.02	0.498	0.80	740.71	768.99
		Time x Day	2.294	-1.37	5.96	1.84	0.216	0.80	740.71	768.99
7 kHz	Base	Intercept	4.791	2.70	6.88	1.04	0.000	0.80	774.87	786.15
	Model 1	Time	1.004	-0.32	2.33	0.67	0.136	0.80	774.87	786.15
	Model 2	Time	1.005	-0.33	2.34	0.67	0.139	0.80	775.70	789.80
		Day	0.061	-1.37	1.49	0.72	0.932	0.80	775.70	789.80
	Model 3	Time	1.177	-0.62	2.98	0.90	0.197	0.80	775.20	792.12
		Day	0.242	-1.66	2.15	0.96	0.801	0.80	775.20	792.12
		Time x Day	-0.380	-3.05	2.29	1.34	0.777	0.80	775.20	792.12
	Model 4	Time	0.490	-2.26	3.24	1.38	0.723	0.79	769.70	797.91
		Day	-0.401	-3.39	2.58	1.50	0.789	0.79	769.70	797.91
		Sex	-1.316	-5.90	3.27	2.30	0.570	0.79	769.70	797.91
		Time x Day	1.385	-2.97	5.74	2.18	0.528	0.79	769.70	797.91
8 kHz	Base	Intercept	3.840	1.60	6.08	1.11	0.001	0.88	606.81	617.31
	Model 1	Time	-0.462	-1.59	0.67	0.57	0.417	0.88	606.81	617.31
	Model 2	Time	-0.478	-1.61	0.65	0.56	0.401	0.88	606.89	620.01

	Day	-0.631	-1.82	0.55	0.59	0.291	0.88	606.89	620.01
Model 3	Time	-1.328	-2.83	0.18	0.75	0.083	0.88	603.99	619.74
	Day	-1.545	-3.14	0.05	0.80	0.057	0.88	603.99	619.74
	Time x Day	1.907	-0.36	4.17	1.13	0.097	0.88	603.99	619.74
Model 4	Time	-1.168	-3.12	0.79	0.98	0.236	0.89	593.16	619.41
	Day	-2.068	-4.30	0.16	1.11	0.069	0.89	593.16	619.41
	Sex	2.250	-2.49	6.99	2.36	0.345	0.89	593.16	619.41
	Time x Day	4.470	1.23	7.71	1.62	**0.008***	0.89	593.16	619.41

Note. This table presents results from linear mixed-effects models assessing the effects of Time: Morning vs. Afternoon and Day; Beginning of week vs. End of week of the week, and sex on DPOAE amplitudes (3–8 kHz). β represents the estimated effect size, with 95% confidence intervals (CI), standard errors (SE), and intraclass correlation coefficients (ICC). Model fit is evaluated using the Akaike Information Criterion (AIC) and Bayesian Information Criterion (BIC). P-values= **>0.05***

Citations in Appendix

Johnston, R., Jones, K. & Manley, D. Confounding and collinearity in regression analysis: a cautionary tale and an alternative procedure, illustrated by studies of British voting behaviour. *Qual Quant* 52, 1957–1976 (2018). https://doi.org/10.1007/s11135-017-0584-6
