# Supplementary material for: Short-term impact of preschool sound exposure on outer hair cell function in young children: An analysis using pressurised distortion product otoacoustic emissions
Source: PLoS One. 2025 Nov 21;20(11):e0332863. doi: 10.1371/journal.pone.0332863 (PMC12637916; doi:10.1371/journal.pone.0332863)

Diagnostic Plots

To evaluate the assumptions underlying the linear mixed-effects models, we conducted a series of diagnostic checks. Supplemental S4–S15 Figs display Q-Q plots and Residuals vs. Fitted plots for all significant and non-significant models. These plots confirm the normality of residuals and the homoscedasticity of variances, which are critical for the validity of the model results. No substantial deviations were observed, providing confidence in the robustness of the findings reported in the main manuscript.

Figure A. QQ-plots and Residuals vs. Fitted values for Right Ear 3 kHz

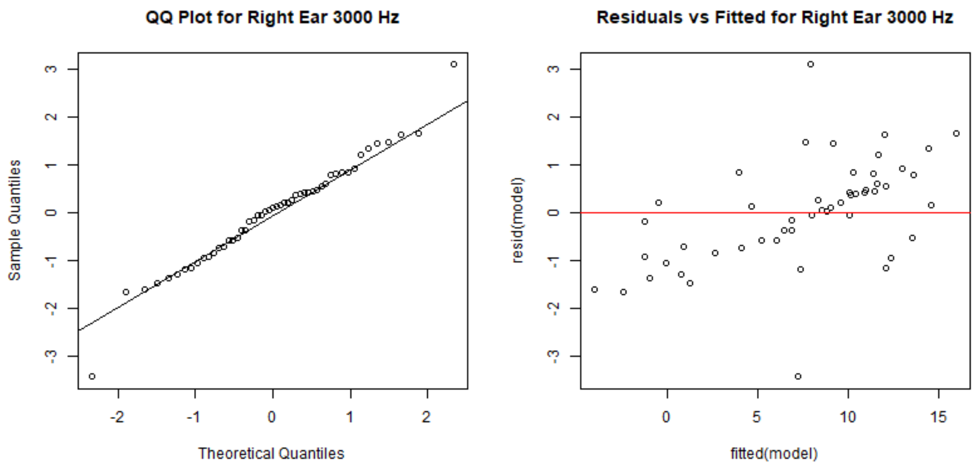

Figure B. QQ-plots and Residuals vs. Fitted values for Right Ear 4 kHz

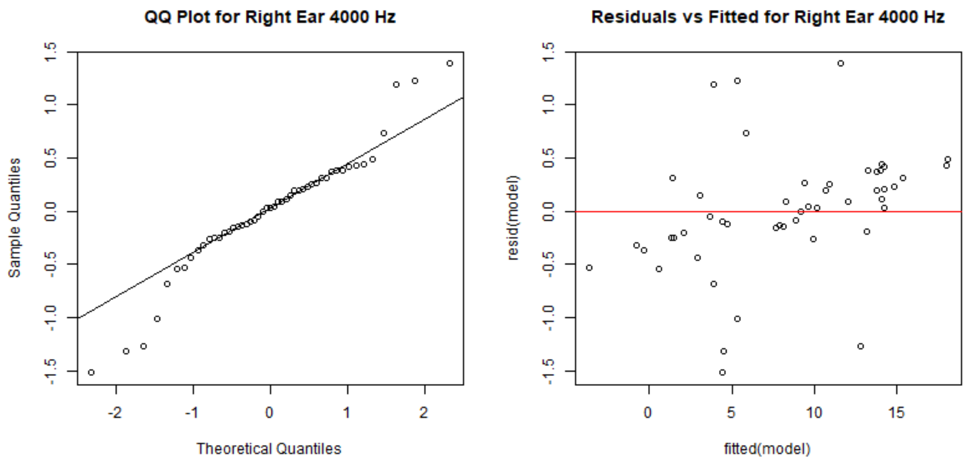

Figure C. QQ-plots and Residuals vs. Fitted values for Right Ear 5 kHz

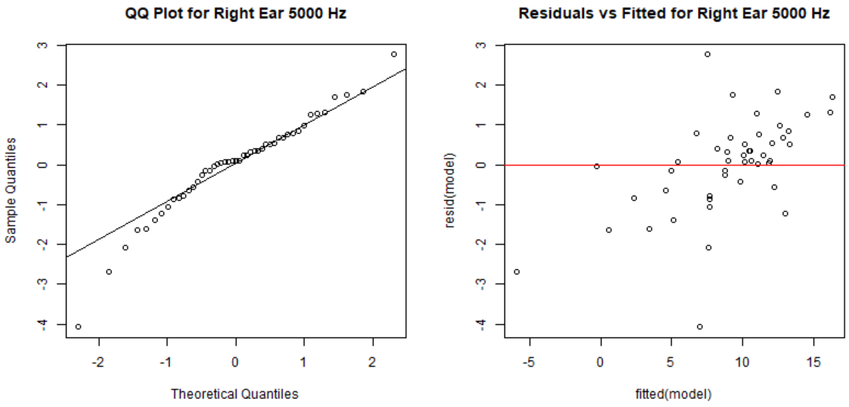

Figure D. QQ-plots and Residuals vs. Fitted values for Right Ear 6 kHz

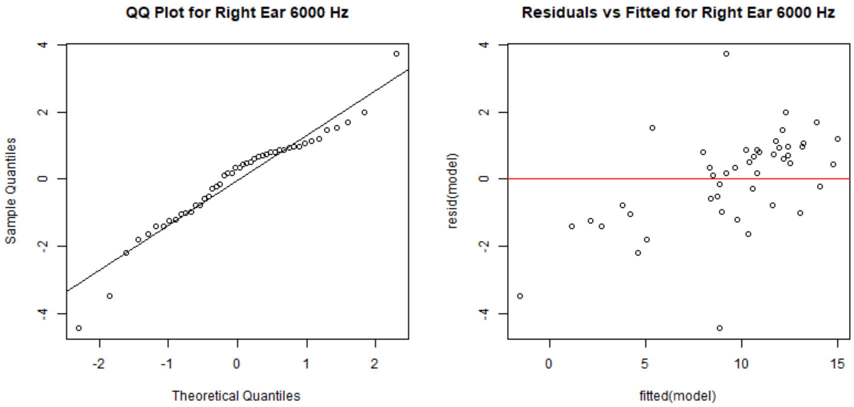

Figure E. QQ-plots and Residuals vs. Fitted values for Right Ear 7 kHz

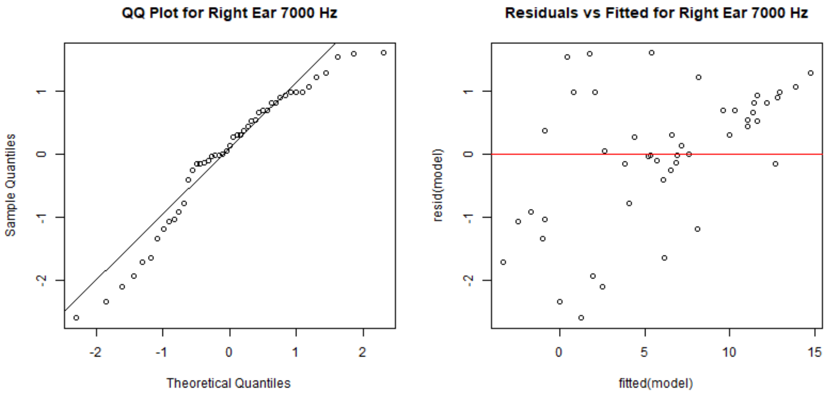

Figure F. QQ-plots and Residuals vs. Fitted values for Right Ear 8 kHz

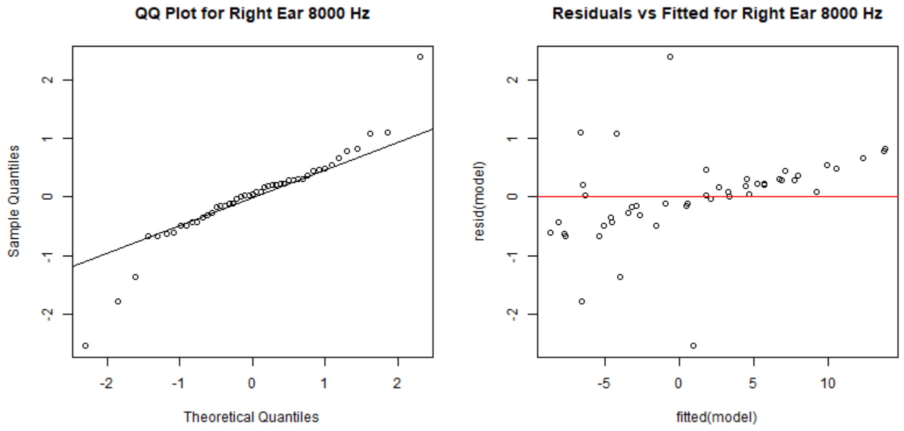

Figure G. QQ-plots and Residuals vs. Fitted values for Left Ear 3 kHz

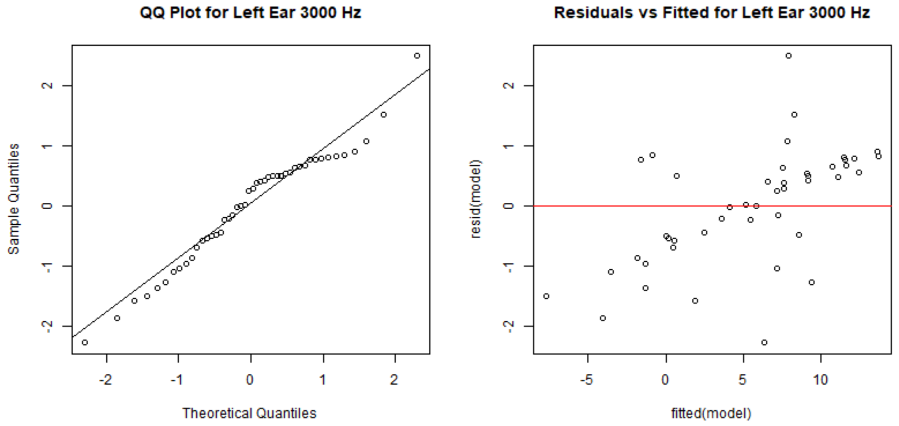

Figure H. QQ-plots and Residuals vs. Fitted values for Left Ear 4 kHz

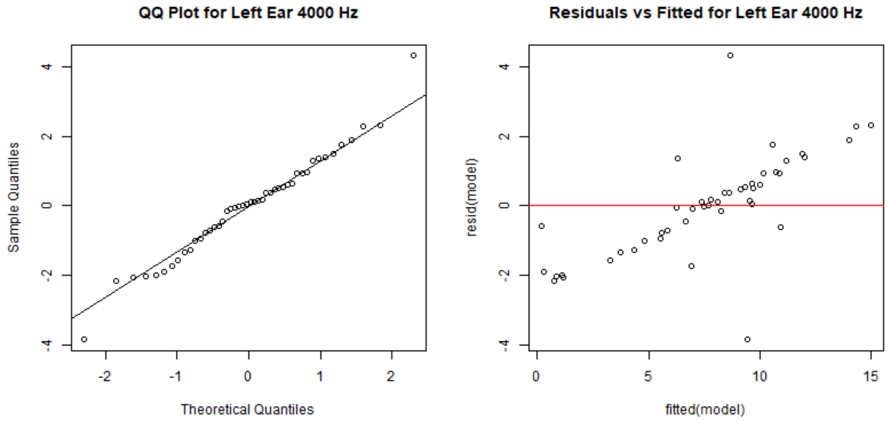

Figure I. QQ-plots and Residuals vs. Fitted values for Left Ear 5 kHz

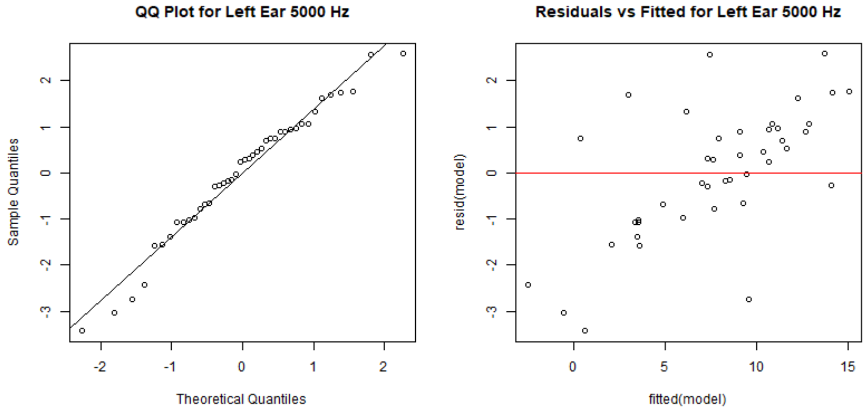

Figure J. QQ-plots and Residuals vs. Fitted values for Left Ear 6 kHz

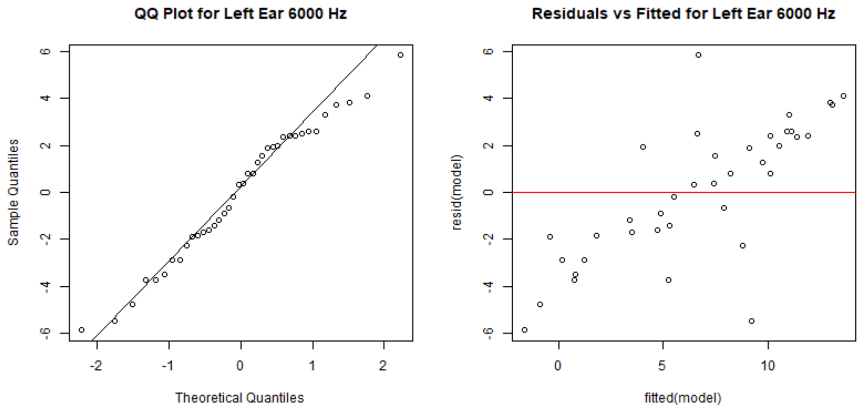

Figure K. QQ-plots and Residuals vs. Fitted values for Left Ear 6 kHz

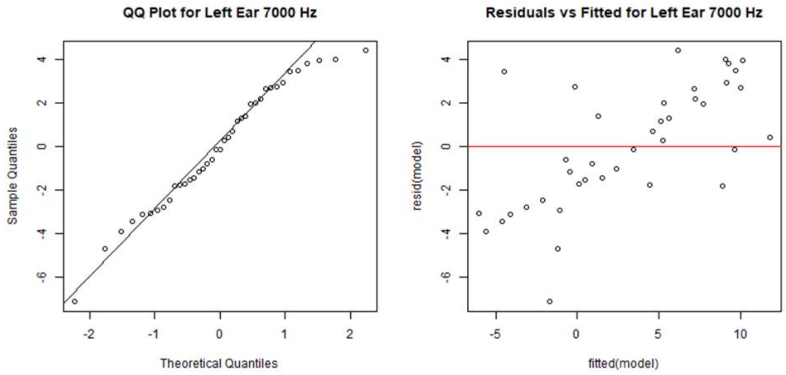

Figure L. QQ-plots and Residuals vs. Fitted values for Left Ear 8 kHz

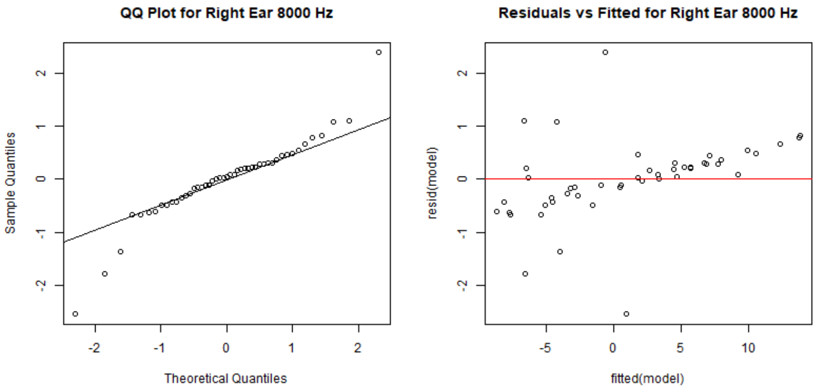

Supplement: S2 File — (PDF) [file pone.0332863.s002.pdf]
