# Supplementary figures and images for: Short-term impact of preschool sound exposure on outer hair cell function in young children: An analysis using pressurised distortion product otoacoustic emissions
Source: PLoS One. 2025 Nov 21;20(11):e0332863. doi: 10.1371/journal.pone.0332863 (PMC12637916; doi:10.1371/journal.pone.0332863)

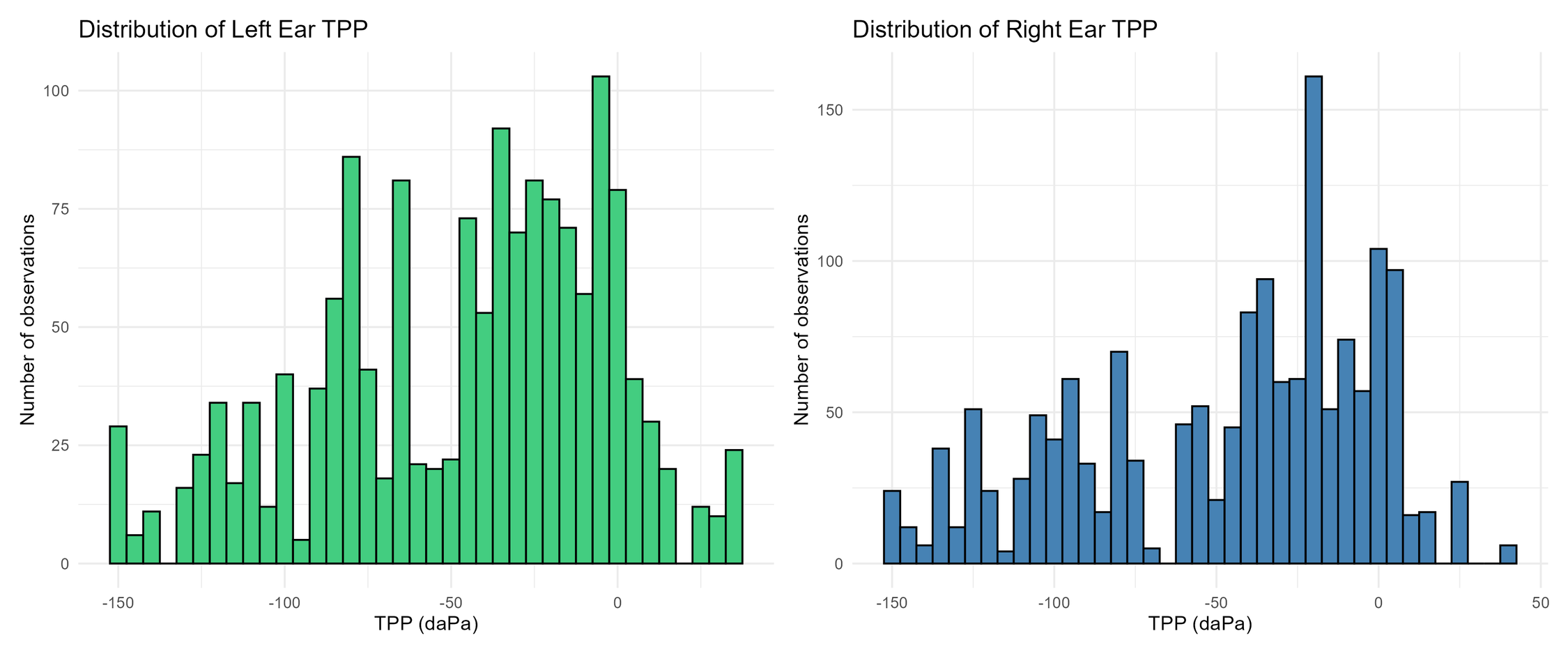

Supplement: S1 Fig — (TIF) [file pone.0332863.s003.tif]

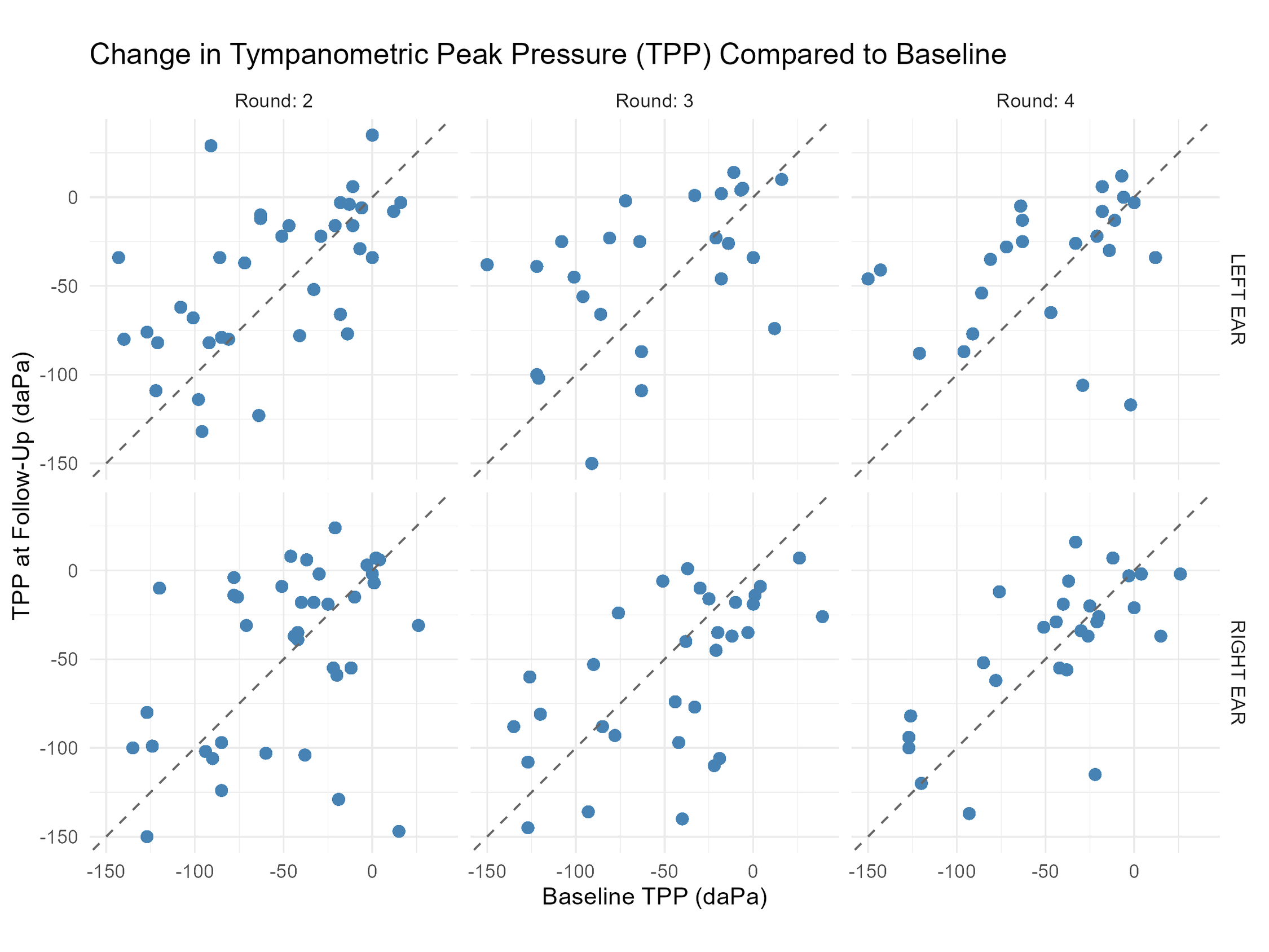

Supplement: S2 Fig — (TIF) [file pone.0332863.s004.tif]

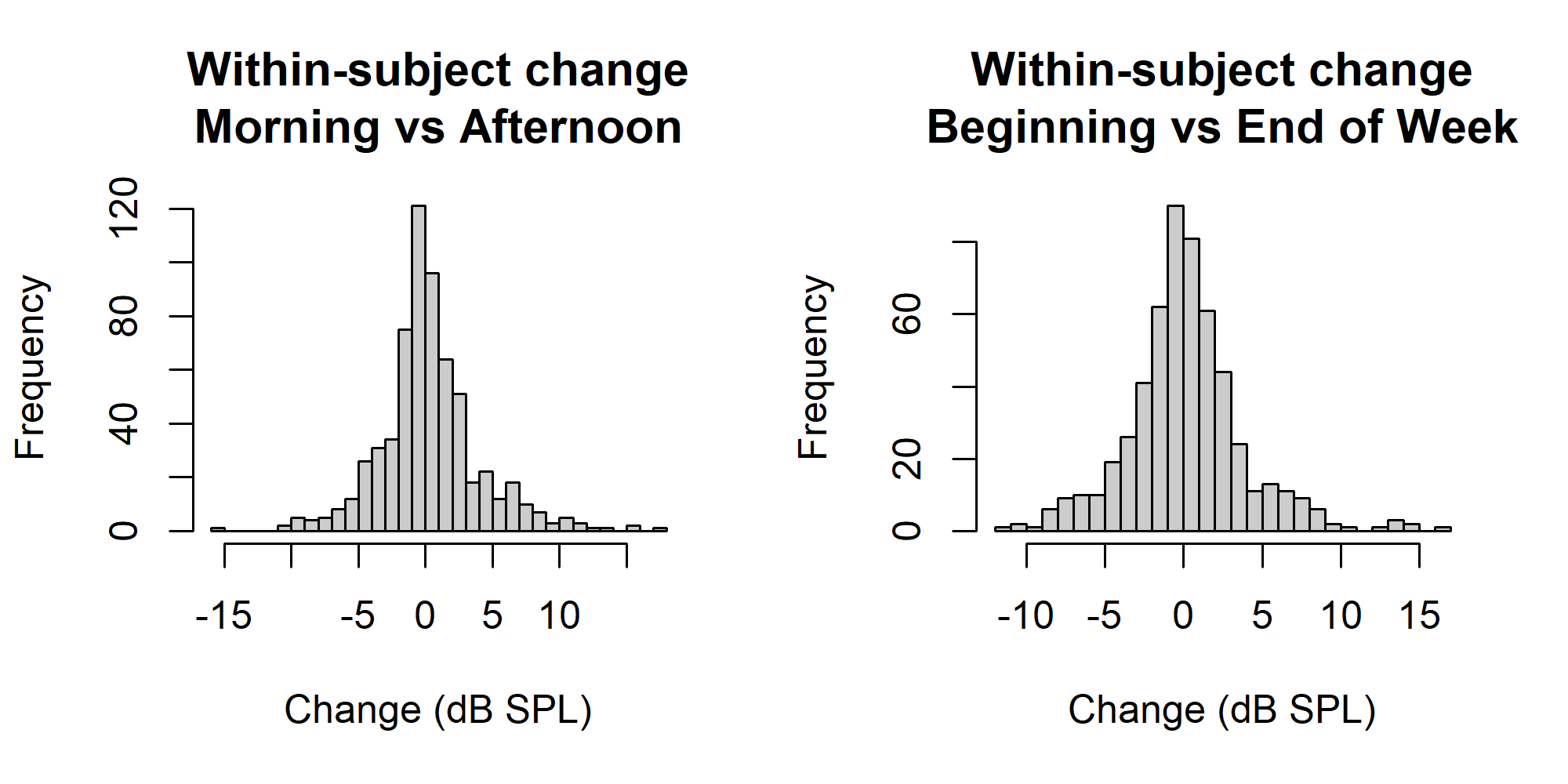

Supplement: S3 Fig — (TIF) [file pone.0332863.s005.tif]
