# Supplementary material for: Short-term impact of preschool sound exposure on outer hair cell function in young children: An analysis using pressurised distortion product otoacoustic emissions
Source: PLoS One. 2025 Nov 21;20(11):e0332863. doi: 10.1371/journal.pone.0332863 (PMC12637916; doi:10.1371/journal.pone.0332863)
Supplement: Attachment — Submitted filename: Rebuttal_Letter.docx [file pone.0332863.s008.docx]

# Response to Reviewer Comments

**Manuscript Title:** *Impact of Preschool Sound Exposure on Hearing Function in Young Children: An Analysis of Pressurised Distortion Product Otoacoustic Emissions*

**UPDATED, Manuscript Title:** *Short-Term Impact of Preschool Sound Exposure on Outer Hair Cell Function in Young Children: An Analysis Using Pressurised Distortion Product Otoacoustic Emissions*

**Manuscript Number:** PONE-D-25-17569R1

**Journal:** PLOS One

**Date:** 20250708

**Corresponding Author:** Loisa Sandström, Loisa.Sandstrom@gu.se

## General Statement

We thank the editor and reviewers for their thoughtful and constructive feedback. We have carefully addressed each point and revised the manuscript accordingly. In addition to responding to all scientific comments, we have made the necessary updates to ensure full compliance with PLOS ONE’s editorial and data policies, including manuscript formatting, author listing, data availability, and Supporting Information captions.

Below, we provide a point-by-point response.

- Section 1 includes responses to requests from the Editorial Office.
- Section 2 contains our detailed replies to each reviewer comment.

In each case, reviewer/editor comments are shown in bold (and as standalone headings in Section 2), followed by our response and a description of any changes made to the manuscript.

**Please note** that all line numbers referenced in this rebuttal refer to the version of the manuscript with tracked changes **not** visible.

## Editorial Office Requests

We thank the editorial team for these clear and detailed instructions. Below we outline the steps taken to ensure compliance with all editorial and data requirements:

**1. Manuscript Formatting**
The manuscript file has been reviewed and revised to follow PLOS ONE's style requirements, including formatting and file naming conventions, as per the provided templates: *Manuscript Body Formatting Guidelines* and *Title, Author, Affiliations Formatting Guidelines*

**2. Data Availability and Ethics**
**Response:**
Thank you for highlighting this important clarification. We have now revised the Data Availability Statement in the manuscript and submission system to comply with PLOS ONE’s data policy. Due to ethical restrictions outlined in our approved protocols (Swedish Ethical Review Authority Dnr: 2019-03412, 2020-03944, 2022-03580-02), we are not permitted to share raw individual-level data on children's hearing and noise exposure publicly.

A minimal anonymised dataset, excluding directly identifiable or sensitive information, will be therefore made available to qualified researchers upon request. The Swedish National Data Service (SND) now serves as the institutional, non-author contact point for these requests. Full contact details and a persistent link to the dataset description will be included upon final archiving. The submission form and manuscript have been updated accordingly.

**Updated Data Availability Statement (for manuscript and submission system)**

The data underlying the results presented in this study cannot be shared publicly owing to ethical restrictions on research involving children, as specified in approvals from the Swedish Ethical Review Authority (Dnr 2019-03412, 2020-03944, 2022-03580-02). A minimal anonymised dataset (excluding directly identifiable or sensitive information) has been deposited with the Swedish National Data Service (SND) and is available to qualified researchers who meet the criteria for access to confidential data. The dataset is available at https://doi.org/10.5878/kp2z-2239. For further enquiries, or to request access to the data, email [request@snd.se](mailto:request@snd.se).

**3. Author Addition**
Co-author Kerstin Persson Waye has been added in the online submission system with appropriate authorship contributions. We have carefully reviewed both the manuscript and the submission metadata to ensure all co-authors are listed correctly. If there is still an oversight on our part, we would be grateful for clarification and will promptly make any necessary corrections.

**4. Supporting Information Captions**
Captions for all Supporting Information files (S1–S15 Figures and S1–S7 Tables) have now been added at the end of the manuscript. All in-text citations have been reviewed and updated to ensure consistency with the corresponding Supporting Information file labels and order.

We hope that these updates address all outstanding requirements and are happy to provide further clarification if needed.

# Reviewers Comments

# Reviewer 1:

## 1.1 Line 30: DPOAE should be in parentheses here. ¨

**Response:**
Corrected in the revised manuscript.

## 1.2 Line 37: pDPOAE or DPOAE?

**Response:**
This has been corrected as it should be pDPOAE, thank you for pointing this out.

## 1.3 Line 265 and 298: Here (and on 298), military time is used but on line 171, it is not. Pick one and stay consistent through the manuscript.

**Response:**
Thank you. Time formatting has been standardised to “military time”, 24h clock, throughout the manuscript.

## 1.4 Line 434: I thought the p in pDPOAE stood for pressurised, not processed.

**Response:**
The wording has been corrected to pressurised.

## 1.5 Table 4: Remove the ‘i’ in measurements.

**Response:**
This has been corrected.

## The introduction is well-written and lays out the purpose for conducting this study well.

**Response:** Thank you!

## 1.6 Line 203 Hearing Assessment Section: pDOAEs are not well known. Please explain in more detail how it compensates for middle ear pressure variations. Additionally, please indicate for the readers unaware of DPOAEs what higher amplitudes suggest compared to low (e.g., good OHC function above the noise floor).

**Response:**
Thank you for this helpful comment. We have expanded the background with regards to the Methods section (Lines 231–235) to further explain pressurised DPOAEs (pDPOAEs).

To address the second part of your comment, we added an explanation in the Discussion (Lines 535–537). We have also added a new section introducing DPOAEs more clearly in the Introduction (Lines 119–129) and expanded the Introduction (Lines 143–149) to provide further context on pDPOAE and its methodological advantages.

## Line 277-278: data excluded for lunchtime and outdoor periods. Is there also a “quiet time” or “nap time” that was also accounted for?

**Response:**

Thank you for this important observation. Nap time was not a factor in this dataset, as the participating departments catered to older preschool children who did not have scheduled naps. Instead, the early afternoon typically involved calmer activities. These periods were not excluded, as they reflect the natural variation in preschool routines. To clarify this point, we have added information the Methods section (Lines 308–310).

## 1.7 Throughout the manuscript, the acronym changes between pDPOAE and DPOAE. Please review all instances and make sure they are reporting the correct one.

Thank you. We have reviewed the manuscript and ensured consistent, correct use of *DPOAE* and *pDPOAE* throughou

## 1.8 Line 350: Which citation are you referencing for these interpretation values of ICC?

**Response:**

Thank you for pointing this out, the correct reference has been added to the manuscript (on line 382):

Koo, T. K., & Li, M. Y. (2016). A Guideline of Selecting and Reporting Intraclass Correlation Coefficients for Reliability Research. Journal of chiropractic medicine, 15(2), 155–163. <https://doi.org/10.1016/j.jcm.2016.02.012¨>

1.9 Figure 4: Include r and p values in the figure (usually top left) for each ear

**Response:**
We thank the reviewer for this helpful suggestion. Figure 4 has been updated accordingly.

## 1.10 Line 427 & 440: I am confused here. It is reported that there are no associations but then later is says there are potential associations. Please clarify.

**Response:**

Thank you for this helpful observation. We agree that the original wording in the figure legend could cause confusion between visual trends in the scatter plots and the main inferential results. In the previous version, “potential associations” referred to the visual pattern of points and the descriptive Pearson r values shown in Figure 4, not to statistically significant effects. As stated in the Results, inferential statistics from the linear mixed models did not identify significant associations between individual sound exposure and pDPOAE amplitudes. We have therefore revised the figure legend (Lines 480–490) to make this distinction explicit, clarifying that the Pearson correlations are presented for descriptive purposes only and that the main results are based on linear mixed models.

## 1.11 Line 457/462/468: significantly higher means better here, correct? Please include interpretation.

**Response:**

Thank you for this important comment. Yes, higher DPOAE amplitudes are generally interpreted as reflecting better outer hair cell responsiveness. In line with reporting conventions, we have kept the Results section strictly descriptive and have therefore not included this interpretation there. In order to address your point, we now explicitly state in the Discussion (Lines 533–537) that higher amplitudes typically indicate better OHC responsiveness, whereas reductions are generally interpreted as consistent with temporary changes in OHC function. This interpretation is also supported by the revised Introduction (Lines 124–129), which notes that DPOAE amplitudes can decrease following noise exposure, indicating a temporary reduction in OHC function that may suggest early signs of cochlear stress.

## 1.12 Line 538: add the referenced study citation here.

**Response:**

## Thank you for noting this omission. Please note that the section where the reference originally appeared has been revised following other reviewer comments and no longer appears in its previous form, the citation appears on the lines: 595, 612, 638

## The correct citation is:

## Thompson S, Henin S, Long GR. Negative middle ear pressure and composite and component distortion product otoacoustic emissions. Ear Hear. 2015;36: 695–704. doi:10.1097/AUD.0000000000000185

## 1.4 Line 570: Please explain why A-weighting was used given these limitations. Either here or in the Methods section.

**Response:**

Thank you for this important comment. We agree that clarification is needed. A-weighting was used because it remains the internationally recognised standard for environmental and occupational noise measurement and is widely used in studies of preschool environments. While we acknowledge that A-weighting is based on adult hearing sensitivity and may not fully capture how young children perceive sound it enables comparability with previous research and supports alignment with regulatory thresholds and guidelines. This rationale has now been added to the Discussion section on lines 685-687.

## 1.13 Future Directions: It would also be interesting to see if there is a relationship between personal dosimetry/DPOAE and teacher performance reports. That is, are those with higher dosimetry results also those that have more behavioral or attention problems seen by the teacher?

**Response:**
Thank you for this thoughtful suggestion. While formal teacher evaluations are not part of the pedagogical mission in Swedish preschools, we did collect behavioural data using the Strengths and Difficulties Questionnaire (SDQ), completed by preschool staff. These data are part of a broader longitudinal study and will be analysed in relation to other environmental factors, including room acoustics and longer-term exposure patterns.

In the present study, our focus was limited to the short-term effects of noise exposure on cochlear function, using pDPOAE as a physiological outcome measure. Including behavioural outcomes such as those from the SDQ would require a different analytical framework and longer follow-up. We agree that a broader perspective is important and consider the SDQ data a valuable resource for future work within the longitudinal project.

# Reviewer 2

Major points:
2.1 The use of pDPOAEs can reduce the confound of negative middle ear pressure primarily at frequencies ≤2 kHz (Beck et al., 2016, Hear Rev). However, the authors only analyzed pDPOAEs from 3-8 kHz, so the advantage of using pDPOAEs over standard (unpressurized) DPOAEs would expected to be minimal. A limitation of this study is the lack of a control condition using standard DPOAEs. This would allow the authors to directly compare standard vs. pDPOAEs to determine if there is an advantage of pDPOAEs for the current study.

**Response:**
We appreciate this thoughtful comment and agree that the benefits of pressurised DPOAEs (pDPOAEs) are most pronounced at lower frequencies (≤ 2 kHz). However, our focus on the 3–8 kHz range was intentional, as these higher frequencies are more sensitive to early noise-induced cochlear changes, which aligns with the study’s aim to investigate potential short-term effects of preschool noise exposure on outer hair cell function. Although Sun and Shaver (2009) reported that the amplitude-enhancing effect of positive middle-ear pressure was greatest at ≤ 2 kHz, they also observed measurable improvements at 3 kHz, indicating that middle-ear status can still influence responses in this range.

A direct comparison between standard and pressurised DPOAEs was beyond the intended scope of this study. Our objective was to implement a robust, field-feasible protocol optimised for young children. Given the high prevalence of mild negative middle-ear pressure in this population, pressurisation was applied across all tested frequencies to minimise variability between sessions, even at higher frequencies where the benefit is smaller. Importantly, previous studies have not reported adverse effects of pressurisation at higher frequencies, supporting its uniform application in the present protocol. We have clarified this rationale and justification in the revised Discussion (lines 599–609).

## 2.2 DPOAE amplitudes decrease as middle ear pressure becomes more negative (e.g., Thompson et al., 2015, Ear Hear). The authors excluded participants with tympanometric peak pressures falling outside of +50 to -150 daPa, but they did not report the tympanometric peak pressures of the included participants. Therefore, the extent to which pDPOAEs were advantageous in this study cannot be determined. If most participants had tympanometric peak pressures near 0 daPa, the results of pDPOAEs should be similar to standard (unpressurized) DPOAEs.

**Response:**

We thank the reviewer for raising this point. The revised Results (lines 446–452) now report detailed tympanometric peak pressure (TPP) data for all included recordings. Nearly all TPP values were between –100 and +50 daPa, with about half in the –49 to –1 daPa range (see S3 Table; Supplementary Fig. S1). Thus, a substantial proportion of sessions exhibited mild-to-moderate negative middle-ear pressure, where unpressurised DPOAEs would likely have been attenuated. For sessions with TPP near 0 daPa, pDPOAE and standard DPOAE would yield comparable results.

We also included paired baseline and follow-up TPP values for each child and ear in Fig. S2, showing that many individual measurements differed between the two timepoints on the same day. These patterns indicate that negative middle-ear pressure was common in this cohort, further supporting the decision to use pDPOAEs to reduce variability. These new analyses and rationale are now reflected in the Methods (lines 392–397), Results (lines 446–452), and Discussion (lines 598–602).

## 2.3 The authors found some differences in pDPOAE amplitudes across time. However, there were no measurements of immediate test-retest reliability of pDPOAEs. Therefore, it is unclear if these differences in amplitude fell within measurement variability or represent true changes in cochlear function.

**Response:**

We thank the reviewer for highlighting this issue. Immediate test–retest was not undertaken. Pressurised DPOAE in 4–6-year-olds requires tympanometry, pressure matching and stable probe placement. Adding immediate repeats would extend sessions and increase participant burden in a field protocol that already included up to four measurements per child in one week. This falls outside the study’s objective, which was to examine short-term changes in relation to real-world exposure. We now state this explicitly in the Discussion (lines 614–622¨).

# Minor points:

## 2.4 The Introduction lacks sufficient background on DPOAEs (e.g., how they are generated and measured) and the differences between pDPOAEs and DPOAEs.

**Response:**

We thank the reviewer for this helpful suggestion. We have revised the Introduction on lines 119–129. These revisions are supported by additional foundational and contemporary references (Kemp, 1978; Probst et al., 1991; Hall, 2016). pDPOAE is also described more closely under Method, lines 262-264.

## 2.5 Throughout the manuscript, the authors refer to the pDPOAEs as measures of "hearing function." This is not accurate because pDPOAEs are a physiologic measure of outer hair cell function and not a measure of auditory perception. Also, pDPOAE frequencies should be referred to as "f2 frequency" rather than "hearing frequency" (e.g., the x-axis label of Fig. 3).

**Response:**

We thank the reviewer for this important observation and fully agree that pDPOAEs are physiological measures of outer hair cell function, not direct indicators of auditory perception or overall hearing function. In response, we have revised the manuscript to ensure that terminology is used more precisely throughout. References to “outer hair cell function” are now applied when describing the specific mechanism assessed by pDPOAEs, while “cochlear function” is used in broader contexts, such as when discussing overall cochlear status or middle-ear effects. The term “auditory function” is reserved for more general or developmental considerations and is only used when clearly distinguished from physiological outcomes.

The manuscript title has also been updated to reflect this distinction, now specifying “outer hair cell function” as the main outcome: **Short-Term Impact of Preschool Sound Exposure on Outer Hair Cell Function in Young Children: An Analysis Using Pressurised Distortion Product Otoacoustic Emissions**

In addition, in line with the reviewer’s suggestion, we have revised Figure 3 and related text so that frequency is now consistently referred to as “f₂ frequency” in accordance with DPOAE convention. These changes ensure the terminology throughout the manuscript is both accurate and scientifically appropriate.

## 2.6 L434: It appears that "processed" should be replaced with "pressurized." Consider clarifying.

**Response:**

Thank you we have corrected this.

2.7 L435: It is unclear how pDPOAE amplitudes can be expressed in units of dB(A). Consider clarifying.

**Response:**
We thank the reviewer for identifying that the pDPOAE amplitude values were mistakenly labelled as dB(A) instead of the correct unit, dB SPL (sound pressure level). This has now been corrected in the legend of Figure 4 (line 483).

2.8 L437-438: Note that the left panel of Fig. 4 shows the right ear results and the right panel shows the left ear results.

**Response:**

This has been revised for clarity. The figure legend now reads:
*“Panels are ordered right ear first, then left ear.”* to clearly reflect the panel orientation and avoid confusion.

2.9 L530: Note that Tables S3-S5 are referenced in the text before Table S2.

**Response:**
Thank you for noting this inconsistency. We have revised the numbering and order of the supplementary materials to ensure alignment with their first appearance in the main text. Additionally, we have inserted a new Table S3 and two new supplementary figures (S1, S2 and S3) and updated the numbering of all supplementary items accordingly. We have also reviewed the entire manuscript to confirm that all supplementary tables, figures, and main manuscript items are now cited in the correct sequential order.

# Reviewer 3

General Comments

3.1 The authors conducted a study that has important implications for early detection of sound induced auditory dysfunction in young children. The methodology includes several strengths including dosimetry documentation of the duration and dose of noise exposure, the use of a highly sensitive measure cochlear (outer hair cell) function (DPOAEs), and a DPOAE measurement technique that accounts for middle ear pressure as defined by tympanometry. There are several serious weaknesses in the study methodology including limited baseline information about the auditory status of participants and the brief (one week) period of data collection. The authors might consider a similar study that eliminates these weaknesses, and that focuses on an older (adolescent, teenage, and/or college) student population.

**Response:**

We thank the reviewer for recognising the methodological strengths of the study and its relevance for early detection of auditory dysfunction in young children. We acknowledge the limitations noted, including the lack of full baseline audiological profiles and the brief, one-week data collection period. These design choices were intentional, as the study aimed to examine short-term changes in outer hair cell function in response to typical preschool noise exposure, rather than long-term outcomes. This age group was deliberately selected due to their heightened vulnerability and underrepresentation in noise research, despite practical and ethical constraints on testing. We have clarified the study aims to emphasise the focus on short-term, real-world exposure and ecological validity (Methods, lines 177–181; Future Directions, lines 761–763, 770–772; Conclusion, lines 809–812), and we now explicitly discuss the absence of detailed baseline audiometry and time-linked out-of-school exposures as limitations. Baseline screening and exclusion criteria, including repeated tympanometry and otoscopy to maximise valid inclusion, are described in the Methods (lines 194-199).

Specific Comments

3.2 More detailed information about the study participants should be provided. For example, did any of the children have perinatal risk factors for auditory, and specifically cochlear, dysfunction (e.g., admission to a neonatal intensive care unit, perinatal infection, ototoxic drugs, family history)?

**Response:**
We appreciate the reviewer’s interest in more detailed participant information. This study was designed to examine short-term variation in cochlear responses among typically developing preschool children without known risk factors for auditory dysfunction. Hearing status and medical history were systematically assessed via caregivers report and strict exclusion criteria lines 194–200.

## 3.3 Was pure tone and speech audiometry performed and, if so, what were the findings? If not, why wasn’t hearing status fully evaluated before or at the beginning of the study?

**Response:**

We thank the reviewer for raising this important point regarding hearing status assessment. Pure tone and speech audiometry were not performed in this study. This decision was based on both methodological and practical considerations. Our participants were aged 4–6 years, and reliable psychoacoustic audiometric testing is rarely feasible in this age group under field conditions, as such tests require a sound-attenuated environment and sustained cooperation. The only Swedish speech audiometry test validated for research use (HINT-C) is validated only from age 6 to 11. and could therefore not be used for most participants.

Given the primary aim, to detect short-term changes in outer hair cell function following sound exposure, DPOAE provides the most sensitive and objective assessment available in this age group. As they can detect subtle or transient cochlear changes that may not be identified by pure tone audiometry and do not require behavioural responses, it was the choice for this field study.

To address the risk of undetected hearing pathology, we implemented a multi-step screening protocol. Caregivers were asked to confirm the absence of known hearing impairment, ear pathology, or recent illness prior to inclusion. At each measurement occasion, all children underwent otoscopic examination and tympanometry to exclude active ear disease or middle-ear dysfunction. Only children meeting all screening criteria were included for pDPOAE measurement. Following the exclusion from tympanometric screening, the remaining data was evaluated for signal quality and was only kept if they fell within the predefined thresholds.

Methods section more clearly outline the data inclusion criteria (lines 314–328) And Line 194-196 has been rewritten to more clearly state that: *” Children were excluded if there was parent-reported hearing impairment, a history of ear disease or middle-ear surgery, middle-ear dysfunction as determined by tympanometry, or any acute illness on the test day (e.g., colds, flu, ear pain).”*

## 3.4 Was there any attempt to document the participants’ exposure to noise or music outside of school hours, e.g., the child’s use of personal sound devices?

**Response:**
Thank you for this important comment. As part of the consent form, we also collected general information on participants’ listening habits outside of school hours, such as use of personal audio devices and frequency of being asked to lower the volume, via a caregiver questionnaire at the time of consent. Most children either never or only occasionally used headphones or were asked to reduce the volume at home; very few reported daily use. However, these background data were collected solely to provide descriptive context, were not time-linked to the measurement period, and were not used in the primary analyses.

For this reason, they were not presented with the main results. We have clarified in the Discussion (lines 579–591) that the absence of detailed, time-specific data on out-of-preschool noise and music exposure is a limitation, and that future studies should incorporate more precise, contemporaneous assessment of such exposures to better account for total auditory load.

## 3.5 The authors mention in the Discussion section the possibility of “selection bias.” The rationale should be explained for exclusion criteria that involved DPOAE findings, e.g., “Measurements were excluded if the DPOAE amplitude was below −10 dB SPL, if the signal-to-noise ratio was less than 6 dB, or if measurement reliability was below 80%”. It would be useful to know how many potential participants were excluded because of these criteria. Is it possible that children who are susceptible to noise or music induced cochlear dysfunction were excluded from the study because of these criteria or, as the authors state, "skewing the ... samples toward children with more stable physiology"?

**Response:**

We thank the reviewer for raising this point. We have revised the Methods (lines 322–328), Results (lines 454–461), and Discussion (lines 648–660) to clarify the rationale for exclusion criteria, their application, and implications for sample characteristics.

No participants were excluded from the study based on DPOAE criteria; all enrolled children remained in the dataset. However, individual pDPOAE measurements were excluded at the ear–frequency level if they did not meet predefined quality thresholds (amplitude < –10 dB SPL, SNR < 6 dB, or reliability < 80%), applied only after tympanometric screening confirmed middle-ear status.

As detailed in Supplementary Tables S1 and S2 and reported in the Results (lines 441–444), most exclusions were due to tympanometry rather than pDPOAE criteria. For example, at 5–6 kHz in the right ear, 78–80 datapoints were excluded due to tympanometry, while only an additional 14–23 were excluded for pDPOAE-specific thresholds. A similar pattern was observed for the left ear.

This approach minimised artefactual variability from middle-ear dysfunction but may have led to underrepresentation of children with more variable cochlear or middle-ear status. As stated in the revised Discussion (lines 662-667), this methodological trade-off favours validity over full data retention, and we now discuss this as a limitation for external validity rather than using the term “selection bias.”

3.6 Visual analysis of possible DPOAE changes from the beginning to the end of the week (Figure 3) would be simplified if right versus left ear data were plotted on separate graphs, and composite DPOAE amplitudes recorded at the beginning versus end of the week were represented with different colors and plotted on the same graph. The authors may also consider another similar figure but with DPOAE amplitudes for the beginning versus the end of the week displayed as a scatter plot.

Response:

We thank the reviewer for this constructive suggestion. We have implemented the primary recommendations by separating right and left ear data into separate panels and using distinct colours to distinguish beginning and end of week measurements on the same graph (Figure 3, lines 463–469). We believe this revised format provides a clear visual representation of patterns across frequencies for both ears. The additional scatter plot was considered but not included, as we judged the current format to adequately address the reviewer’s main request.

3.7 Exclusive analysis of group data may obscure possible noise effects for individual participants. Did any of the participants show meaningful changes in DPOAE amplitudes over time?

Response:

We thank the reviewer for this question. The study was designed for group-level analysis. No formal statistical test for individual change was undertaken. Descriptive inspection of individual pDPOAE trajectories indicated that while some children showed amplitude differences exceeding typical short-term variability reported in paediatric OAE literature, these patterns were inconsistent across frequencies and sessions. Given the limited sample size per child and the absence of established clinical cut-offs for pressurised measurements in this age group, such individual changes cannot be interpreted as definitive evidence of noise effects. The manuscript therefore focuses on aggregated results, which align with the study’s primary aim of assessing group-level changes in relation to real-world exposure.

Future directions 772-777

## 3.8 The authors state in the Discussion section that “This study investigated how real-world preschool noise exposure relates to cochlear function in young children”. However, potential effects of classroom noise on cochlear function are likely to be chronic, occurring over months and years rather than days. Why were data collected only over a one-week time frame, rather than over the course of a semester or a school year? The authors raise this possibility in the Discussion section: “It is also possible that effects occurred prior to the measurement session or require longer follow-up periods cochlear function in young children to be captured.” The authors should consider further discussion of this important point.

**Response:**
Thank you for highlighting this important point. The present study was specifically designed to assess whether short-term variability in preschool noise exposure, both within days and across a typical week, was associated with measurable changes in cochlear function. The one-week protocol was selected to examine early, potentially reversible changes in outer hair cell function (Discussion, lines 575–579).

We further note (lines 589–591) that future studies should include precise, time-specific measures of out-of-preschool noise and music exposure to enable a more comprehensive assessment of total auditory load. The limitation in the absence of detailed, time-specific data on out-of-school noise or music exposure limits our ability to fully account for all sources of potential variance in cochlear outcomes during the study period is also brough tup in discussion, lines 579-583.

The need for longer-term or repeated measurement designs is explicitly discussed in the Future Directions section, which highlights (lines 760–764) the ethical constraints on experimental research in this age group and recommends longitudinal observational studies to assess cumulative effects. We also state that longitudinal research will be crucial to understanding cumulative effects on cochlear function over time (lines 769--771) and emphasise (lines 808–811) the importance of tracking cochlear function across early childhood to determine whether repeated exposure influences hearing development in the long term.

To further emphasise the intended scope of the study, we have also revised the paper title and clarified the study aim to explicitly state that we investigated short-term effects of noise exposure on children’s cochlear function. These clarifications make the scope and limitations of our findings explicit, and we have revised the manuscript throughout to avoid overstating the implications of the short-term data.
