# Supplementary material for: Short-term impact of preschool sound exposure on outer hair cell function in young children: An analysis using pressurised distortion product otoacoustic emissions
Source: PLoS One. 2025 Nov 21;20(11):e0332863. doi: 10.1371/journal.pone.0332863 (PMC12637916; doi:10.1371/journal.pone.0332863)
Supplement: Attachment — Submitted filename: reviewer comments.docx [file pone.0332863.s006.docx]

**Title**: Impact of Preschool Sound Exposure on Hearing Function in Young Children: An Analysis of Pressurised Distortion Product Otoacoustic Emissions

**Line 30:** DPOAE should be in parentheses here.

**Line 37:** pDPOAE or DPOAE?

The introduction is well-written and lays out the purpose for conducting this study well.

**Line 203 Hearing Assessment Section:** pDOAEs are not well known. Please explain in more detail how it compensates for middle ear pressure variations. Additionally, please indicate for the readers unaware of DPOAEs what higher amplitudes suggest compared to low (e.g., good OHC function above the noise floor).

**Line 265:** Here (and on 298), military time is used by on line 171, it is not. Pick one and stay consistent through the manuscript.

**Line 277-278:** data excluded for lunchtime and outdoor periods. Is there also a “quiet time” or “nap time” that was also accounted for?

Throughout the manuscript, the acronym changes between pDPOAE and DPOAE. Please review all instances and make sure they are reporting the correct one.

**Line 350:** Which citation are you referencing for these interpretation values of ICC?

**Table 4**: remove the ‘i’ in measurements

**Figure 4:** Include r and p values in the figure (usually top left) for each ear

**Line 434:** I thought the p in pDPOAE stood for pressurized not processed

**Line 427 & 440:** I am confused here. It is reported that there are no associations but then later is says there are potential associations. Please clarify.

**Line 457/462/468:** significantly higher means better here, correct? Please include interpretation.

**Line 538:** add the referenced study citation here.

**Line 570:** Please explain why A-weighting was used given these limitations. Either here or in the Methods section.

**Future Directions:** It would also be interesting to see if there is a relationship between personal dosimetry/DPOAE and teacher performance reports. That is, are those with higher dosimetry results also those that have more behavioral or attention problems seen by the teacher?
